# Supplementary material for: Cellular mechanisms of heterogeneity in NF2-mutant schwannoma
Source: Nat Commun. 2023 Mar 21;14:1559. doi: 10.1038/s41467-023-37226-0 (PMC10030849; doi:10.1038/s41467-023-37226-0)
Supplement: Supplementary file 3 — Description of Additional Supplementary Files [file 41467_2023_37226_MOESM3_ESM.pdf]

Description of Additional Supplementary Files

File Name: Supplementary Movie 1
Description: Phase contrast timelapse imaging showing results of collision between WT SCs. Video of Figure 2 E visualizing cells that were plated and allowed to adhere for 15 minutes before unbound cells were washed away. Frames were captured every 5 minutes and displayed at 7 frames per second.

File Name: Supplementary Movie 2
Description: Phase contrast timelapse imaging showing results of collision between $Nf2^{-/-}$ SCs. Video of Figure 2 E visualizing cells that were plated and allowed to adhere for 15 minutes before unbound cells were washed away. Frames were captured every 5 minutes and displayed at 7 frames per second.
