# Supplementary material for: Cellular mechanisms of heterogeneity in NF2-mutant schwannoma
Source: Nat Commun. 2023 Mar 21;14:1559. doi: 10.1038/s41467-023-37226-0 (PMC10030849; doi:10.1038/s41467-023-37226-0)
Supplement: Supplementary file 6 — Reporting Summary [file 41467_2023_37226_MOESM6_ESM.pdf]

nature portfolio

Corresponding author(s): Andrea I. McClatchey, Ph.D

Last updated by author(s): 02/23/2023

Reporting Summary

Nature Portfolio wishes to improve the reproducibility of the work that we publish. This form provides structure for consistency and transparency in reporting. For further information on Nature Portfolio policies, see our Editorial Policies and the Editorial Policy Checklist.

Statistics

For all statistical analyses, confirm that the following items are present in the figure legend, table legend, main text, or Methods section.

n/a	Confirmed	
☐	☒	The exact sample size (*n*) for each experimental group/condition, given as a discrete number and unit of measurement
☐	☒	A statement on whether measurements were taken from distinct samples or whether the same sample was measured repeatedly
☐	☒	The statistical test(s) used AND whether they are one- or two-sided<br>*Only common tests should be described solely by name; describe more complex techniques in the Methods section.*
☒	☐	A description of all covariates tested
☐	☒	A description of any assumptions or corrections, such as tests of normality and adjustment for multiple comparisons
☐	☒	A full description of the statistical parameters including central tendency (e.g. means) or other basic estimates (e.g. regression coefficient) AND variation (e.g. standard deviation) or associated estimates of uncertainty (e.g. confidence intervals)
☐	☒	For null hypothesis testing, the test statistic (e.g. *F*, *t*, *r*) with confidence intervals, effect sizes, degrees of freedom and *P* value noted<br>*Give P values as exact values whenever suitable.*
☒	☐	For Bayesian analysis, information on the choice of priors and Markov chain Monte Carlo settings
☒	☐	For hierarchical and complex designs, identification of the appropriate level for tests and full reporting of outcomes
☐	☒	Estimates of effect sizes (e.g. Cohen's *d*, Pearson's *r*), indicating how they were calculated

Our web collection on statistics for biologists contains articles on many of the points above.

Software and code

Policy information about availability of computer code

Data collection	Zeiss Zen (black edition) software (version 2.1) was used to collect the confocal fluorescence images. Akoya Biosciences Vectra software (version 3.0.5) and Phenochart whole slide contextual viewer software (version 1.1) were used to capture whole slide scans of human and mouse tissue. Akoya Biosciences InForm software (version 2.4.1) was used for spectral unmixing of whole slide scans of human and mouse tissue. Roche Applied Science LightCycler 480 Software (version 1.5.1) was used to collect quantitative RT-PCR data.
Data analysis	ImageJ software (version 2.0, National Institutes of Health) was used for image analysis. HALO digital image analysis software version 3.4 (Indica Labs) was used to analyze whole slide scans of mouse and human tumor samples.

For manuscripts utilizing custom algorithms or software that are central to the research but not yet described in published literature, software must be made available to editors and reviewers. We strongly encourage code deposition in a community repository (e.g. GitHub). See the Nature Portfolio guidelines for submitting code & software for further information.

Data

Policy information about availability of data

All manuscripts must include a data availability statement. This statement should provide the following information, where applicable:

- Accession codes, unique identifiers, or web links for publicly available datasets
- A description of any restrictions on data availability
- For clinical datasets or third party data, please ensure that the statement adheres to our policy

The data that support the findings of this study are available in the article or the supplementary information files. Source data is available in the source data file provided with this paper.

Human research participants

Policy information about studies involving human research participants and Sex and Gender in Research.

Reporting on sex and gender

Tumor samples (Vestibular schwannoma) from both females and males were included in the study. This information was collected by the tumor bank (UCLA Brain Tumor Translational Resource) but is not known by the authors of the study. Sex was not considered as a variable in the study since there are no known differences between females and males in the physiopathology of neurofibromatosis type 2.

Population characteristics

Tumor samples (Vestibular schwannoma) were collected and anonymized from sporadic and neurofibromatosis type 2 patients during resection surgery by the UCLA Brain Tumor Translational Resource.

Tumor number	Tumor type	Patient
16008-9	Vestibular schwannoma	NF2
16009-9	Vestibular schwannoma	NF2
16011-9	Vestibular schwannoma	NF2
13104-9	Vestibular schwannoma	Sporadic

Recruitment

Vestibular schwannoma samples were randomly chosen among sporadic and neurofibromatosis type 2 patients in the tumor database.

Ethics oversight

UCLA Institutional Review Board (IRB), protocol #10-000655.

Note that full information on the approval of the study protocol must also be provided in the manuscript.

Field-specific reporting

Please select the one below that is the best fit for your research. If you are not sure, read the appropriate sections before making your selection.

☒ Life sciences ☐ Behavioural & social sciences ☐ Ecological, evolutionary & environmental sciences

For a reference copy of the document with all sections, see nature.com/documents/nr-reporting-summary-flat.pdf

Life sciences study design

All studies must disclose on these points even when the disclosure is negative.

Sample size

No statistical method was used to predetermine sample size. Sample sizes were made as large as possible given the constraints of the experiment. All image-based cell data was analyzed using individual cells as the experimental unit. For all experiments, at least 3 cells were measured in 3-10 images randomly captured across the coverslip, which we have found to be sufficient to meet statistical significance across many previous studies in the lab. For RT-PCR analysis each sample was run in triplicate and at least 2 independent experiments were conducted. For studies of human tissues, tumors from 4 patients were analyzed. For studies of mouse tissues, at least 12 DRG from each mouse were analyzed.

Data exclusions

No data were excluded from the analyses.

Replication

Repetitive biologically independent experiments were conducted to confirm consistency of results. For all analyses at least 2, and in most cases 3 independent experiments were conducted to verify the reproducibility of the experimental findings. All replication attempts were successful. Sample sizes and number of biologically independent experiments with consistent results are indicated in the corresponding figure legends.

Randomization	For studies of human tissues, vestibular schwannoma samples were randomly chosen among sporadic and neurofibromatosis type 2 patients in the tumor database. For studies of mouse tissues, groups (e.g. Nrg+, TGFa+) were determined using the multiplex IHC or FISH-IF algorithms in HALO software to identify positively stained cells at single cell resolution. For studies in cell lines comparisons were made between defined groups (e.g. WT vs. Nf2-/- or control vs. drug treatment) and randomization was not required.
Blinding	Investigators were not blinded to experimental conditions during data collection. Data collection for fluorescent confocal microscopy imaging was conducted through the selection of random fields of view and analysis parameters were not obvious to the human eye. For this reason the experimenters were inherently blinded to the outcome of the experiment and further measures were not needed. For analysis of mouse and human tumors, HALO software algorithms were used for high throughput analysis of all tumor cells, making blinding unnecessary. Blinding during data analysis was not possible because all data was analyzed by the first or second author.

Reporting for specific materials, systems and methods

We require information from authors about some types of materials, experimental systems and methods used in many studies. Here, indicate whether each material, system or method listed is relevant to your study. If you are not sure if a list item applies to your research, read the appropriate section before selecting a response.

Materials & experimental systems

n/a	Involved in the study	
☐	☒	Antibodies
☐	☒	Eukaryotic cell lines
☒	☐	Palaeontology and archaeology
☐	☒	Animals and other organisms
☒	☐	Clinical data
☒	☐	Dual use research of concern

Methods

n/a	Involved in the study	
☒	☐	ChIP-seq
☒	☐	Flow cytometry
☒	☐	MRI-based neuroimaging

Antibodies

Antibodies used	The following primary antibodies were used: anti-ezrin mouse monoclonal antibody (mAb) (1:500; clone 3C12, #MA5-13862, Invitrogen); anti-p75 NTR rabbit mAb (1:1000; clone D8A8, #4201, Cell Signaling Technology); anti-N-cadherin mouse mAb (1:500; clone 32, #610921, BD Biosciences); anti-beta-catenin mouse mAb (1:500; clone 14, #610154, BD Biosciences); anti-pAkt (S473) rabbit mAb (IF: 1:100, WB: 1:1000; clone D9E, #4060, Cell Signaling Technology); anti-Akt rabbit mAb (1:1000; clone 11E7, #4685, Cell Signaling Technology); anti-phospho p44/42 (ERK1/2) rabbit mAb (1:1000; clone D13.14.4E, #4370, Cell Signaling Technology); anti-p44/42 (ERK1/2) rabbit pAb (1:1000; #9102, Cell Signaling Technology); anti-ErbB2 rabbit pAb (IF: 1:100; A0485, Agilent), anti-ErbB3 mouse mAb (IF: 1:100, WB: 1:1000; clone RTJ2, #MA1-860, Invitrogen); anti-E-cadherin mouse mAb (1:1000; clone 36, #610182, BD Biosciences); anti-actin mouse mAb (1:1000; clone AC-40, #A4700, Millipore Sigma); anti-merlin rabbit mAb (1:100; clone D1D8, #6995, Cell Signaling Technology); anti-pS6 (S235/236) rabbit pAb (1:100; #2211, Cell Signaling Technology); anti-Nrg1 rabbit pAb (1:100; #ab191139, Abcam); anti-TGFa rabbit pAb (1:100; #ab9585, Abcam); anti-pNDRG1 rabbit mAb (1:100; clone D98G11, #5482, Cell Signaling Technology); anti-Neurofilament L rabbit mAb (1:100; clone C28E10, #2837, Cell Signaling Technology). The following secondary antibodies were used for IF at a dilution of 1:500: goat anti-rabbit IgG (H+L) highly cross-adsorbed secondary antibody, Alexa Fluor 488 (#A-11034, Invitrogen); goat anti-rabbit IgG (H+L) highly cross-adsorbed secondary antibody, Alexa Fluor 555 (#A-21428, Invitrogen); goat anti-rabbit IgG (H+L) highly cross-adsorbed secondary antibody, Alexa Fluor 647 (#A-21244, Invitrogen); goat anti-mouse IgG (H+L) highly cross-adsorbed secondary antibody, Alexa Fluor 488 (#A-11001, Invitrogen); goat anti-mouse IgG (H+L) highly cross-adsorbed secondary antibody, Alexa Fluor 555 (#A-21422, Invitrogen); and goat anti-mouse IgG (H+L) highly cross-adsorbed secondary antibody, Alexa Fluor 647 (#A-21235, Invitrogen). For western blotting, ECL anti-Rabbit IgG, horseradish peroxidase-linked whole antibody (from donkey) (#GENA934, Millipore Sigma) and ECL anti-mouse IgG, horseradish peroxidase-linked whole antibody (from sheep) (#GENA931, Millipore Sigma) were used at a dilution of 1:5000.
Validation	anti-ezrin (clone 3C12, MA5-13862): The manufacturer's website states that this antibody has been tested for WB, IHC, IF, and IP, and has species reactivity for human, mouse, dog, and rat. The antibody was verified by knockdown to ensure that the antibody binds to the antigen stated (Invitrogen).<br>anti-p75NTR (clone D8A8, #4201): The manufacturer's website states that this antibody detects endogenous levels of total p75NTR protein. It recognizes and unidentified protein at 42 kDa. Validated by WB and has species reactivity for human, mouse, and rat (Cell Signaling Technology).<br>anti-N-cadherin (clone 32, #610921): The manufacturer's website states that this antibody has been tested for WB and IF and has species reactivity for human, mouse, rat, and chicken (BD Biosciences).<br>anti-beta-catenin (clone 14, #610154): The manufacturer's website states that this antibody has been tested for WB, IHC, IF, and IP and has species reactivity for human, mouse, rat, dog, and chicken (BD Biosciences).<br>Phospho-Akt (Ser473) (clone D9E, #4060): The manufacturer's website states that this antibody detects endogenous levels of Akt1 only when phosphorylated at Ser473. This antibody also recognizes Akt2 and Akt3 when phosphorylated at the corresponding residues. Validated by WB in wortmannin-treated PC-3 cells. Tested for WB, IHC, IF, flow cytometry, and IP and has species reactivity for human, mouse, rat, hamster, monkey, D. melanogaster, and bovine. (Cell Signaling Technology).<br>anti-Akt (clone 11E7, #4685): The manufacturer's website states that this antibody has been tested for WB, IHC, IF, flow cytometry, and IP and has species reactivity for human, mouse, rat, and monkey (Cell Signaling Technology).<br>Phospho-p44/42 MAPK (Erk1/2) (Thr202/Tyr204) (clone D13.14.4E, #4370): The manufacturer's website states that this antibody detects endogenous levels of p44 and p42 MAP Kinase (Erk1 and Erk2) when dually phosphorylated at Thr202 and Tyr204 of Erk1 (Thr185 and Tyr187 of Erk2), and singly phosphorylated at Thr202. This antibody does not cross-react with the corresponding phosphorylated residues of either JNK/SAPK or p38 MAP kinases. Validated by western blot in U0126-treated COS cells. Tested for

WB, IHC, IF, flow cytometry, and IP and has species reactivity for human, mouse, rat, hamster, monkey, mink, D. melanogaster, zebrafish, dog, pig, s. cerevisiae, and bovine. (Cell Signaling Technology).
anti-p44/42 (ERK1/2) (#9102): The manufacturer's website states that this antibody has been tested for WB and IP and has species reactivity for human, mouse, rat, hamster, monkey, mink, zebrafish, pig, bovine, and s. cerevisiae. (Cell Signaling Technology).
anti-ErbB2 (A0485): The manufacturer's website states that this antibody was tested by WB and has species reactivity for human and mouse. (Agilent)
anti-ErbB3 (clone RTJ2, #MA1-860): The manufacturer's website states that this antibody was verified by relative expression to ensure that the antibody binds to the antigen stated. Tested for WB, IHC, IF, flow cytometry, ELISA, and IP and has species reactivity for human and mouse (Invitrogen).
anti-actin (clone AC-40, #A4700): The manufacturer's website states that this antibody has been tested for WB, IHC, IF, and ELISA and has species reactivity for human, mouse, rat, hamster, guinea pig, Xenopus, rabbit, snail, sheep, dog, carp, pig, bovine, viper, and goat. Millipore Sigma)
anti-E-cadherin (clone 36, #610182): The manufacturer's website states that this antibody was validated by western blot in control 293F cells (no expression) and 293F cells transfected with human E-cadherin. Cross reactivity with P-cadherin was noted. Tested for WB, IHC, IF, and IP and has species reactivity for human, mouse, rat, and dog (BD Biosciences).
anti-merlin (cloneD1D8, #6995): The manufacturer's website states that this antibody was tested by WB and IP and has species reactivity for human, mouse, and rat. The antibody was validated in supplementary Figure 3B by IF staining of Nf2-/- and Nf2WT-expressing schwannoma cells.
Phospho-S6 Ribosomal Protein (Ser235/236) (#2211): The manufacturer's website states that this antibody detects endogenous levels of ribosomal protein S6 only when phosphorylated at serine 235 and 236. This antibody does not detect ribosomal protein S6 phosphorylated at other sites. Tested for WB, IHC, IF, flow cytometry, and IP and has species reactivity for human, mouse, rat, monkey, and s. cerevisiae. (Cell Signaling Technology).
anti-Nrg1 (#ab191139): The manufacturer's website states that this antibody was tested by WB, IF, and IHC in frozen and formalin fixed parafin-embedded sections and has species reactivity for human and rat (predicted to work with mouse) (Abcam).
anti-TGFa (#ab9585): The manufacturer's website states that this antibody was tested by WB, IHC, and ELISA and has species reactivity for human and mouse (Abcam).
Phospho-NDRG1 (Thr346) (clone D98G11, #5482): The manufacturer's website states that this antibody detects endogenous levels of NDRG1 when phosphorylated at Thr346. This antibody likely cross-reacts with other conserved phosporylation sites on NDRG1 at positions Thr356 and Thr366. Validated by knockdown in HeLa cells. Tested for WB, IHC, IF, and flow cytometry has species reactivity for human, mouse, rat, and monkey (Cell Signaling Technology). Validated for IF: PMID: 24687281 and IHC: PMID: 27758884.
anti-Neurofilament L (clone C28E10, #2837): The manufacturer's website states that this antibody was tested by WB, IHC and IF and has species reactivity for human, mouse, and rat. (Cell Signaling Technology).

Eukaryotic cell lines

Policy information about cell lines and Sex and Gender in Research

Cell line source(s)	Primary murine SCs were isolated from sciatic nerves of Nf2flox/flox adult mice and purified by magnetic sorting. The mouse schwannoma cell line was derived from a tumor dissected from a P0-CreB;Nf2flox/flox mouse and cells were isolated by standard dispase/collagenase dissociation techniques. 293T human embryonic kidney cells containing SV40 T-antigen were obtained from ATCC (#CRL-3216). Ad-293 human embryonic kidney cells were obtained from Agilent (#240085).
Authentication	Primary murine SCs were authenticated using STR profiling through ATCC's Cell Line Authentication Service.
Mycoplasma contamination	All cell lines tested negative for mycoplasma contamination.
Commonly misidentified lines (See ICLAC register)	This study did not use any commonly misidentified cell lines.

Animals and other research organisms

Policy information about studies involving animals; ARRIVE guidelines recommended for reporting animal research, and Sex and Gender in Research

Laboratory animals

Mouse strain	Symbol	MGI ID	Genetic background
Postn-Cre	Tg(Postn-cre)1Sjc	MGI:3775923	Backcrossed >10 times in FVB/N
Nf2flox	Nf2tm2Gth	MGI:1926955	Backcrossed >10 times in FVB/N

Postn-Cre;Nf2flox/flox mice were used at 6 and 12 months of age. Both female and male mice were included in the study.
Dark/light cycle: 12 hours of light/12 hours of dark.
Ambient temperature range: 68-79F (20-26C)
Ambient humidity range: 30-70%

Wild animals	This study did not involved wild animals.
Reporting on sex	Both female and male mice were included in the study. Sex was not considered as a variable in the study since there are no known differences between females and males in the physiopathology of neurofibromatosis type 2.
Field-collected samples	This study did not involved samples collected from the field.
Ethics oversight	UCLA institutional animal care and use committee (IACUC), protocol #2019-042.

Note that full information on the approval of the study protocol must also be provided in the manuscript.
